# Assembling and validating data from multiple sources to study care for Veterans with bladder cancer

**DOI:** 10.1186/s12894-017-0271-x

**Published:** 2017-09-06

**Authors:** Florian R. Schroeck, Brenda Sirovich, John D. Seigne, Douglas J. Robertson, Philip P. Goodney

**Affiliations:** 1 White River Junction VA Medical Center, 215 N Main Street, White River Junction, VT 05009 USA; 2 Section of Urology, Dartmouth Hitchcock Medical Center, Lebanon, NH USA; 3 Norris Cotton Cancer Center, Dartmouth Hitchcock Medical Center, Lebanon, NH USA; 4 The Dartmouth Institute for Health Policy and Clinical Practice, Geisel School of Medicine at Dartmouth College, Hanover, NH USA

**Keywords:** Bladder cancer, Cystoscopy, Electronic health record, Validity

## Abstract

**Background:**

Despite the high prevalence of bladder cancer, research on optimal bladder cancer care is limited. One way to advance observational research on care is to use linked data from multiple sources. Such big data research can provide real-world details of care and outcomes across a large number of patients. We assembled and validated such data including (1) administrative data from the Department of Veterans Affairs (VA), (2) Medicare claims, (3) data abstracted by tumor registrars, (4) data abstracted via chart review from the national electronic health record, and (5) full text pathology reports.

**Methods:**

Based on these combined data, we used administrative data to identify patients with newly diagnosed bladder cancer who received care in the VA. To validate these data, we first compared the diagnosis date from the administrative data to that from the tumor registry. Second, we measured accuracy of identifying bladder cancer care in VA administrative data, using a random chart review (*n* = 100) as gold standard. Lastly, we compared the proportion of patients who received bladder cancer care among those who did versus did not have full text bladder pathology reports available, expecting that those with reports are significantly more likely to receive care in VA.

**Results:**

Out of 26,675 patients, 11,323 (42%) had tumor registry data available. 90% of these patients had a difference of 90 days or less between the diagnosis dates from administrative and registry data. Among 100 patients selected for chart review, 59 received bladder cancer care in VA, 58 of which were correctly identified using administrative data (sensitivity 98%, specificity 90%). Receipt of bladder cancer care was substantially more common among those who did versus did not have bladder pathology available (96% vs. 43%, *p* < 0.001).

**Conclusion:**

Merging administrative with electronic health record and pathology data offers new possibilities to validate the use of administrative data in bladder cancer research.

**Electronic supplementary material:**

The online version of this article (10.1186/s12894-017-0271-x) contains supplementary material, which is available to authorized users.

## Background

Bladder cancer is the third and fourth most prevalent non-cutaneous cancer among men and women in the United States [1]. In spite of this high prevalence, there is fairly limited research on what entails optimal bladder cancer care [2], particularly for the majority of patients who are living with non-muscle invasive bladder cancer (NMIBC). This may be due to the fact that examining bladder cancer care using observational data often represents a “moving target” [3]. Specifically, patients with bladder cancer tend to have multiple recurrences and after each recurrence their pathology and consequently their bladder cancer risk-classification can change [4, 5], impacting further treatment recommendations and follow-up [3].

One potential way to advance observational research on care for NMIBC is to use linked data from multiple sources to gain a more complete picture of the care patients receive and of the outcomes of that care. Sources may include administrative data from hospital electronic health records (EHR), claims data from Medicare, full text records (e.g. pathology reports) from the EHR, as well as data manually abstracted by chart review. Linking multiple sources into larger, combined datasets – sometimes called big data research [6] – provides the opportunity to capture procedures performed, details of pathology at time of diagnosis and at time of recurrence, and clinical details that can be abstracted from the patient chart, thus providing a more complete picture of patient care and outcomes. Moreover – as done here – these combined datasets allow for the validation of algorithms and results from the administrative data.

Here, we combine multiple data sources from the Department of Veterans Affairs (VA) Corporate Data Warehouse (CDW) to assemble a data set that can be used to study care for patients with bladder cancer. We describe an algorithm to identify patients with newly diagnosed bladder cancer who received bladder cancer care in VA and then examine its convergent, criterion, and concurrent validity. These validated data will provide the opportunity for future detailed research examining utilization and outcomes of surveillance care among patients with bladder cancer.

## Methods

### Data sources

We assembled and linked data from five distinct sources in order to provide a comprehensive picture of bladder cancer diagnosis, pathology, and care. This included (1) administrative data from the VA CDW (including both inpatient and outpatient encounter data), (2) Medicare claims data for the Veterans in our cohort, (3) data abstracted by tumor registrars at each individual VA facility which is then deposited into the CDW, (4) full text pathology reports from the Text Integration Utility files available in the CDW, and (5) data abstracted via chart review from the national electronic health record using the Compensation and Pension Records Interchange (CAPRI) and Veterans Health Information Systems and Technology Architecture (VistA) Web tools.

The VA Information Resource Center (VIReC) routinely obtains Medicare claims data for Veterans and matches these to VA data using established algorithms, based on social security number, gender, and date of birth [7, 8]. Medicare data for members of our cohort were then provided by VIReC. Medicare claims data, tumor registry data, and full text pathology data were linked using the scrambled social security number, a unique patient identifier created for research purposes by VIReC. Data abstracted via chart review were linked using the real social security number. The study was approved by the Dartmouth Committee for the Protection of Human Subjects (#28417) and by the Veteran’s Institutional Review Board of Northern New England (#897920-1).

### Algorithm to identify patients with newly diagnosed bladder cancer

We developed an algorithm based on administrative data to identify a cohort of Veterans with newly diagnosed bladder cancer. For this, we first identified any patient 66 years of age or older with a diagnosis code for bladder cancer (ICD9 codes 188.x, 233.7, 236.7, 239.4) within the VA CDW outpatient and inpatient files between 01/01/2005 and 12/31/2011. For the outpatient files, we required at least two diagnosis codes for bladder cancer at least 30 days apart to help exclude “rule-out” type diagnoses (e.g. a patient with a lesion seen on CT scan prompting a diagnosis code for bladder cancer but later cystoscopy failing to show a tumor within the bladder) [9]. We defined the first occurrence of a bladder cancer diagnosis date as the index date. Next, we excluded any patients with a preexisting diagnosis of bladder cancer (ICD9 188.x, 233.7, V10.51) within the VA CDW inpatient or outpatient data, or within the Medicare Provider Analysis and Review (MEDPAR), Medicare Outpatient, or Medicare Carrier files during the 365 days prior to the index date. Medicare data were queried, because approximately half of VA patients also receive care through Medicare [8]. This left us with a cohort of patients who had a diagnosis code for bladder cancer between 2005 and 2011 and did not have any preexisting bladder cancer diagnosis codes. However, a 365 day look back is arbitrary and may be too short for bladder cancer patients as some of them may only undergo follow-up once a year. Thus, we performed sensitivity analyses after excluding patients with a preexisting diagnosis of bladder cancer in the 730 days prior to the index date (*n* = 23,068). Results from these sensitivity analyses were not materially different in direction or effect size compared with those of our main analyses, so only the latter are presented.

### Assessing the date of diagnosis – Convergent validity

Convergent validity is defined as the degree to which an operationalization is similar to (converges on) other operationalizations that it theoretically should be similar to [10]. In our study, we assessed convergent validity by comparing the diagnosis date from the claims algorithm (that is the index date after applying the 365 day look back as described above) to the diagnosis date from the tumor registry among the subset of patient who had tumor registry data available (Table 1). We calculated the proportion of patients who had the same diagnosis date in both sources and whose tumor registry date fell within a +/− 7 day, 30 day, or 90 day window around the algorithm-derived index date. In addition, we calculated the proportion of patients who did not have newly diagnosed bladder cancer, defined as a tumor registry diagnosis date more than 90 days prior to the algorithm-derived date.

**Table 1:** For each type of validity, the question, the comparison, and the rationale for evaluation are shown

Question	Comparison	Rationale
Can we correctly identify the diagnosis date? (Convergent validity)	Diagnosis dates from claims algorithm vs those from tumor registry (*n* = 11,323)	Tumor registry data are deemed most reliable because registrars abstracted data directly from the chart. However, registry data are not available for all patients, necessitating development of a cohort based on administrative data.
Can we accurately identify bladder cancer care received within VA? (Criterion validity)	Bladder cancer care received in VA based on administrative data vs chart review (n = 100)	(1) Assure that algorithm does find all patients who did get bladder cancer care (sensitivity). (2) Assure that patients who were identified as receiving bladder cancer care with the algorithm actually did receive such care (positive predictive value).
If we apply the algorithm to the entire cohort, can we distinguish between groups that are conceptually more or less likely to receive bladder cancer care in VA? (Concurrent validity)	Bladder cancer care received in VA among patients with vs without full text bladder pathology reports available (*n* = 26,675)	Patients with full text bladder pathology reports are highly suspected to have received bladder cancer care in VA. Thus, the proportion receiving bladder cancer care in VA should be significantly higher among patients who have full text bladder pathology reports than among those who have not.

### Assessing receipt of bladder cancer care in VA – Criterion validity

Criterion validity is defined as the performance of an operationalization against some criterion (gold standard) [10]. In our study, we assessed criterion validity by evaluating our ability to identify bladder cancer care in VA based on administrative data against a chart review of 100 randomly selected cases as the gold standard. We defined bladder cancer care as cystoscopy without or with biopsy or transurethral resection. Using established methods [11], we classified patients as receiving bladder cancer care in the VA if they had evidence for these procedures (see Additional file 1) within the VA administrative data between the index date and study end (12/31/2014). To better understand whether we can correctly identify bladder cancer care, we measured the accuracy of the administrative data to differentiate between patients who did versus those who did not receive bladder cancer care in VA (Table 1). For this, we randomly sampled 100 patients out of the entire cohort for a chart review. We used the national electronic health record to review all relevant clinical notes from 1 year prior to the index date to at least 2 years after the index date. Based on this review, we determined whether the patient did or did not receive bladder cancer care in VA. Using the chart review as the gold standard, we then calculated sensitivity, specificity, negative predictive value, positive predictive value, and accuracy of the claims-based algorithm to identify patients who received bladder cancer care in VA. We calculated confidence intervals (CIs) for these measures using a binomial distribution. Finally, we determined the reasons for not identifying bladder cancer care within VA administrative data, using Medicare enrollment and claims data as well as data from the chart review.

### Assessing our ability to distinguish between groups that are conceptually more or less likely to receive bladder cancer care in VA – Concurrent validity

Concurrent validity examines the operationalization’s ability to distinguish between groups that it should theoretically be able to distinguish between [10]. In our case, patients with full text bladder pathology reports were highly suspected to have received bladder cancer care in VA. Thus, our a priori expectation was that receipt of bladder cancer care in VA should be significantly more common among patients who have full text pathology reports than among those who have not (Table 1). As previously described, we identified full text pathology reports based on the report title indicating a pathology report and on presence of at least one of the three keywords “bladder”, “urethra”, or “ureter” within the full text [12]. We then used the chi-squared test to compare the proportion of patients receiving bladder cancer care among those who did versus who did not have pathology reports available.

## Results

**Fig. 1:**
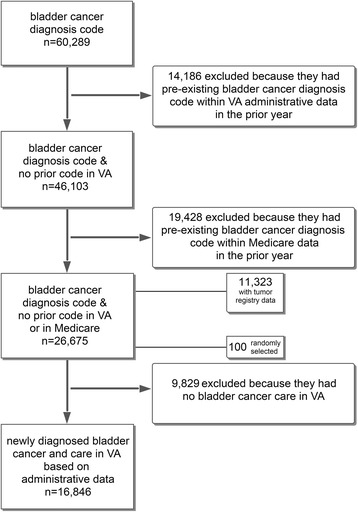
Development of a cohort of patients with newly diagnosed bladder cancer between 2005 and 2011 who received bladder cancer care in VA

The final cohort consisted of 26,675 patients with newly diagnosed bladder cancer, after excluding patients with a pre-existing bladder cancer diagnosis in either VA administrative data or Medicare claims during the 365 days prior to the index date. Approximately two thirds of these patients (*n* = 16,846, 63%) received bladder cancer care in VA (Fig. 1).

### Convergent validity and date of diagnosis

**Fig. 2:**
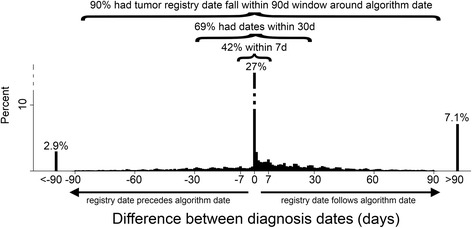
Establishing convergent validity by comparing the diagnosis date obtained from the administrative data to the diagnosis date abstracted by registrars. The histogram shows the percentage of patients that have a given difference between diagnosis dates. The diagnosis date derived from the administrative data (“algorithm date”) was the same as the tumor registry date among 27% of patients. Just 2.9% of patients did not have newly diagnosed bladder cancer, because their registry diagnosis date preceded the date obtained from administrative data by more than 90 days

First, we evaluated whether we can correctly identify the diagnosis date from the administrative data. Thus, we compared the diagnosis date obtained from the administrative data to the diagnosis date abstracted by the registrars among the subset of 11,323 patients (42%) with registry data available. About a quarter of patients (27%) had the same diagnosis date in both datasets. Ninety percent had their tumor registry diagnosis date fall within a 90 day window around the date derived from the administrative data (Fig. 2). Only 2.9% did not have newly diagnosed bladder cancer, as their tumor registry date was more than 90 days prior to the date derived from the administrative data (Fig. 2).

### Criterion validity and receipt of bladder cancer care in VA

Next, we assessed whether we can accurately identify patients who were newly diagnosed with bladder cancer and who received bladder cancer care in the VA based on administrative data. Using the chart review of 100 cases as the gold standard, we were able to differentiate patients who did versus who did not receive bladder cancer care in VA with an accuracy of 95% (95% CI 89% - 98%). All but one patient who had bladder cancer care based on chart review were identified by the use of administrative data (sensitivity 98%, 95% CI 91% - 100%, Table 2). Among the 62 patients who were identified as receiving bladder cancer care with administrative data, 58 actually did receive such care based on chart review (positive predictive value 94%, 95% CI 84% - 98%, Table 2).

**Table 2:** Accuracy of administrative data to identify bladder cancer care in VA among patients with newly diagnosed bladder cancer, based on chart review of 100 randomly selected cases (the gold standard). We were able differentiate between patients who did versus who did not receive bladder cancer care in VA with high accuracy (95%), with only 5 cases having discordant data between chart review and algorithm

Bladder cancer care in VA		Based on chart review (Gold standard)		Total
		Yes	No	
Based on administrative data	Yes	58	4	62
	No	1	37	38
Total		59	41	

Next, we evaluated reasons for not receiving bladder cancer care in VA. The most common reasons included receipt of care outside of the VA among patients who were enrolled in a Medicare HMO, a recent diagnosis of bladder cancer with the patient opting for palliative care only, and a remote history of bladder cancer without any recent recurrences or follow-up care (Additional file 2). We also evaluated the four patients who had no evidence for bladder cancer care in the chart review, but who did have administrative data suggesting such care (Table 2). These patients had unusual specific circumstances, including (1) a remote history of bladder cancer and cystectomy approximately 40 years ago with need for recurrent cystoscopic ureteral stent replacements, (2) a remote history of bladder cancer with subsequent need for cystoscopy with urethral dilation for a urethral stricture, (3) a patient whose prostate cancer diagnosis was miscoded as bladder cancer and who underwent a diagnostic cystoscopy after his prostate cancer treatment, and (4) a patient who had a benign ureteral mass excised and who had a subsequent cystoscopy for stent removal.

### Assessing our ability to distinguish between groups that are conceptually more or less likely to receive bladder cancer care in VA – Concurrent validity

Finally, we assessed whether we can distinguish between groups of patients who should be more or less likely to receive bladder cancer care in VA. Examining the entire cohort, about two thirds of the cohort (16,846 patients, 63%) had bladder cancer care in VA (Fig. 1). We evaluated whether receipt of bladder cancer care was more common among those with full text pathology reports in the database as one would expect. Indeed, the proportion of patients receiving bladder cancer care in VA was significantly higher among those who had full text pathology reports available than among those who had not (96% vs. 43%, Table 3, *p* < 0.001).

**Table 3:** Proportion of patients receiving bladder cancer care in VA based on administrative data, comparing those who did versus who did not have full text bladder pathology reports available. Applying the algorithm to the entire cohort, the proportion receiving bladder cancer care in VA is significantly higher among patients who have full text pathology reports in VA than among those who do not (96% vs 43%, p < 0.001, chi-squared test)

		Bladder cancer care in VA, Percent (N)		Total, Percent (N)
		Yes	No	
Full text pathology available, Percent (N)	Yes	96 (9893)	4 (432)	100 (10,325)
	No	43 (6953)	57 (9397)	100 (16,350)
Total, Percent (N)		63 (16,846)	37 (9829)	100 (26,675)

## Discussion

We combined data from multiple sources to validate the use of administrative data to examine bladder cancer care. Specifically, we provide evidence that we can accurately identify patients with newly diagnosed bladder cancer who received care in the VA healthcare system. Comparison to tumor registry data showed that we can reliably identify an approximate diagnosis date for the vast majority of patients. We also established that we can accurately identify patients who received bladder cancer care in VA. Lastly, we were able to validate that patients who were highly likely to have received bladder cancer care in VA based on the presence of full text bladder pathology reports overwhelmingly also had evidence of that care in administrative data.

Our study leverages advantages conferred by additional data sources including full text pathology reports and chart review of the electronic health record. We were able to identify patients with newly diagnosed bladder cancer who received bladder cancer care in VA with a positive predictive value of 94%, very similar to the 93.8% positive predictive value reported by a study comparing administrative data from a primary care data base in the UK to a survey mailed to primary care physicians as the gold standard [13].

In addition to data obtained by chart review, we also used tumor registry data to validate the algorithm, similar to prior studies [14–17]. Comparing the diagnosis date obtained from the VA administrative data to the tumor registry diagnosis date, we found that about a quarter had the same date in both sources and for 90% the registry date fell within a 90 day window around the date obtained from the administrative data. These findings are very similar to those obtained in a prior study evaluating the accuracy of algorithms to identify newly diagnosed lung, colorectal, stomach, and breast cancers based on SEER-Medicare data (about a quarter had diagnosis dates on the same day and about 90% within a 60 day window) [17].

Our study has several limitations that warrant discussion. First, our approach and findings may not be generalizable to other big data sets from outside the VA. Nevertheless, our study highlights the unique opportunity to use big data on a national scale and may inspire other groups to use similar approaches in their data sets. Second, we were not able to examine the sensitivity of the algorithm to identify patients with bladder cancer. This would have required a national or regional sample of patients with a confirmed bladder cancer diagnosis (regardless of their diagnosis codes) and then application of our algorithm to that cohort to examine whether these patients could be correctly identified. We did not have access to such a cohort, because our institutional review board approval is allowing us only access to patients with a bladder cancer diagnosis code. However, a previous study provided evidence of high sensitivity when identifying bladder cancer patients based on diagnosis codes [13]. Third, we acknowledge that only 42% of patients had tumor registry data available, which appears to be a low proportion. This is likely related to the fact that data is only abstracted among patients who receive their bladder cancer care within the VA. Among those who received their care in VA, the proportion with tumor registry data was substantially higher (9258 of 15,352 patients, 60%). Nevertheless, this relatively low proportion justifies the use of administrative data to assemble a cohort of bladder cancer patients for studies in which it is important to include the entire universe of patients receiving bladder cancer care.

In spite of these limitations, our study has important implications. We highlight the advantages of using big data, that is data from multiple sources merged into one comprehensive data set. This allowed us to validate the use of administrative data as done here and will allows us to develop a more comprehensive understanding of what does and does not matter when providing care for patients with bladder cancer in the future. Previously, the use of observational data to better understand care for patients with early stage bladder cancer has been hampered by the lack of important clinical details in most data sets. Care for early stage bladder cancer has been described as a “moving target” [3], because each patient’s risk for recurrence and progression can change over time. For example, patients who are diagnosed with a low-risk early stage bladder cancer may have a recurrence of a high-risk cancer and vice versa [4, 5, 18]. Standard administrative and tumor registry data do not capture granular data on these recurrences and thus our ability to understand what entails high-quality care for these patients has remained limited. Our comprehensive data set includes administrative data from VA and Medicare, data abstracted by tumor registrars, and full text pathology reports. We expect that this data set validated herein will now make it possible to better understand how bladder cancer care is currently provided and how intensity of cancer care impacts outcomes such as tumor progression and recurrence.

## Conclusions

We demonstrate how merging administrative data with data from the electronic health record, data abstracted by tumor registrars, and pathology data offers new possibilities to validate the use of administrative data. Our validated cohort will now allow us to comprehensively evaluate care and outcomes for patients with bladder cancer.

## Additional files

Additional file 1: Common Procedural Terminology (CPT) codes and International Classification of Diseases (ICD) 9 procedure codes used to identify bladder cancer care (DOCX 19 kb)

Additional file 2: Reasons for not identifying bladder cancer care within VA administrative data among the 100 patients with newly diagnosed bladder cancer randomly selected for chart review (DOCX 18 kb)

## Abbreviations

CAPRI: Compensation and pension records interchange
CDW: Corporate data warehouse
EHR: Electronic health record
MEDPAR: Medicare provider analysis and review
NMIBC: Non-muscle invasive bladder cancer
SEER: Surveillance, epidemiology, and end results
VA: Department of Veterans Affairs
VIReC: VA Information Resource Center
VistA: Veterans Health Information Systems and Technology Architecture
