# Supplementary material for: Assembling and validating data from multiple sources to study care for Veterans with bladder cancer
Source: BMC Urol. 2017 Sep 6;17:78. doi: 10.1186/s12894-017-0271-x (PMC5585934; doi:10.1186/s12894-017-0271-x)
Supplement: Supplementary file 1 — Common Procedural Terminology (CPT) codes and International Classification of Diseases (ICD) 9 procedure codes used to identify bladder cancer care (DOCX 19 kb) [file 12894_2017_271_MOESM1_ESM.docx]

**Additional File 1.** Common Procedural Terminology (CPT) codes and International Classification of Diseases (ICD) 9 procedure codes used to identify bladder cancer care. Cysto = Cystoscopy.

| **Type of Procedure** | **Codes** |
| --- | --- |
| CPT codes | |
| Cysto without or with irrigation, ureteral catheterization, or biopsy | 52000, 52001, 52005, 52007, 52204, 52214 |
| Cysto with urethral dilation or urethrotomy / meatotomy | 52270, 52275, 52276, 52277, 52281, 52282, 52283, 52285, 52290, 52300, 52301, 52305 |
| Cysto for ureteral calculus or stricture | 52320, 52325, 52327, 52330, 52332, 52334, 52341, 52342, 52343 |
| Cysto with transurethral resection of prostate | 52400, 52402, 52450, 52500, 52510, 52601, 52606, 52612, 52614, 52620, 52630, 52640, 52647, 52648, 52649, 52700 |
| Cysto with ureteroscopy | 52344, 52345, 52346, 52351, 52352, 52353, 52354, 52355 |
| Cysto with other intervention | 52250, 52260, 52265, 52310, 52315, 52317, 52318 |
| ICD9 procedure codes | |
| Cysto with biopsy / fulguration | 57.33 |
| Cysto with dilation | 57.91, 57.92 |
| Cysto with transurethral resection of prostate | 60.2, 60.21, 60.29 |
| Cysto with ureteroscopy | 56.31, 56.33 |
| Cysto with or without other intervention | 57.32, 57.39, 57.0 |
