# Supplementary material for: Assembling and validating data from multiple sources to study care for Veterans with bladder cancer
Source: BMC Urol. 2017 Sep 6;17:78. doi: 10.1186/s12894-017-0271-x (PMC5585934; doi:10.1186/s12894-017-0271-x)
Supplement: Supplementary file 2 — Reasons for not identifying bladder cancer care within VA administrative data among the 100 patients with newly diagnosed bladder cancer randomly selected for chart review (DOCX 18 kb) [file 12894_2017_271_MOESM2_ESM.docx]

**Additional File 2.** Reasons for not identifying bladder cancer care within VA administrative data among the 100 patients with newly diagnosed bladder cancer randomly selected for chart review. Among these 100 patients, 38 had no bladder cancer care identified within VA administrative data. For these 38 patients, we used Medicare enrollment and claims data as well as chart review to better understand reasons for not identifying bladder cancer care. HMO = Health Maintenance Organization.

| **Reason** | **Number of Patients** |
| --- | --- |
| VA notes state patient receiving bladder cancer care outside VA and Medicare enrollment data show patient was enrolled in a Medicare HMO | 9 |
| Diagnosed but opted for palliative care only | 9 |
| Only remote history of bladder cancer | 8 |
| Received bladder cancer care through Medicare (based on Medicare claims data) | 5 |
| History of cystectomy and thus no cystoscopy or biopsy received | 4 |
| Care received outside VA but not captured in Medicare for unclear reasons | 2 |
| Miscoded Bladder cancer diagnosis | 1 |
| Total not receiving bladder cancer care in VA | 38 |
